# Efficacy and associated factors of endoscopic transpapillary drainage for postoperative biliary leakage

**DOI:** 10.1002/deo2.281

**Published:** 2023-08-17

**Authors:** Jun Murata, Minoru Shigekawa, Shuji Ishii, Takahiro Suda, Kenji Ikezawa, Motohiro Hirao, Kengo Matsumoto, Tadashi Kegasawa, Kiyoshi Iwahashi, Sadaharu Iio, Fumihiko Nakanishi, Shoichi Nakazuru, Yuichi Yoshida, Takuo Yamai, Katsuhiko Sato, Teppei Yoshioka, Hayato Hikita, Tomohide Tatsumi, Tetsuo Takehara

**Affiliations:** ^1^ Department of Gastroenterology Higashiosaka City Medical Center Osaka Japan; ^2^ Department of Gastroenterology and Hepatology Osaka University Graduate School of Medicine Osaka Japan; ^3^ Department of Gastroenterology and Hepatology Osaka General Medical Center Osaka Japan; ^4^ Department of Gastroenterology and Hepatology Kansai Rosai Hospital Hyogo Japan; ^5^ Department of Hepatobiliary and Pancreatic Oncology Osaka International Cancer Institute Osaka Japan; ^6^ Department of Gastroenterology and Hepatology Osaka Rosai Hospital Osaka Japan; ^7^ Department of Gastroenterology and Hepatology Toyonaka Municipal Hospital Osaka Japan; ^8^ Department of Gastroenterology and Hepatology Ikeda Municipal Hospital Osaka Japan; ^9^ Department of Gastroenterology and Hepatology Osaka Police Hospital Osaka Japan; ^10^ Department of Gastroenterology and Hepatology Hyogo Prefectural Nishinomiya Hospital Hyogo Japan; ^11^ Department of Gastroenterology and Hepatology National Hospital Organization Osaka Minami Medical Center Osaka Japan; ^12^ Department of Gastroenterology and Hepatology National Hospital Organization Osaka National Hospital Osaka Japan; ^13^ Department of Gastroenterology and Hepatology Suita Municipal Hospital Osaka Japan

**Keywords:** biliary fistula, drainage, endoscopic retrograde cholangiopancreatography, post‐cholecystectomy, post‐hepatectomy

## Abstract

**Objective:**

Adequate biliary decompression is important in treating bile leaks, and endoscopic transpapillary drainage is widely used for this purpose. As an indicator to evaluate the usefulness of endoscopic drainage for postoperative biliary leakage, we focused on external drain removability, which affects quality of life, after endoscopic treatment. Our aim was to clarify the success rate of external tube removal after endoscopic drainage for postoperative biliary leakage and to examine associated factors.

**Methods:**

This was a multicenter retrospective study; 99 patients with biliary leakage at 13 institutions were enrolled between April 2014 and March 2019. Among these patients, 66 who were initially treated with endoscopic interventions for biliary leakage after cholecystectomy (*n* = 17) or hepatectomy (*n* = 49) were reviewed.

**Results:**

In post‐cholecystectomy biliary leakage, the external‐drain‐free rate at first endoscopic intervention was 100%, and the drains, including transpapillary stents, were successfully removed in almost all cases (16/17). In contrast, in post‐hepatectomy biliary leakage, the external‐drain‐free rate was 44.9% (22/49), with all 22 of those patients eventually becoming entirely drain‐free. A lower body mass index was the only significant factor associated with freedom from external drainage in post‐hepatectomy biliary leakage (odds ratio 0.18, 95% confidence interval 0.05–0.65).

**Conclusions:**

Initial endoscopic treatment was effective for post‐cholecystectomy biliary leakage, while approximately half of the patients with post‐hepatectomy biliary leakage required multidisciplinary management. Achieving freedom from external drainage contributes to patients’ quality of life and may be a predictor of treatment response after endoscopic therapy for postoperative biliary leakage.

## INTRODUCTION

The incidence of postoperative biliary leakage after laparoscopic cholecystectomy is 0.8%–1.4%, and that after hepatectomy is 3.6%–12.0% without bile duct reconstruction and 0.4%–8.0% with bile duct reconstruction.^1^, ^2^ There are several causes of postoperative biliary leakage, including intraoperative bile duct injury, bile leakage from transected edges, and suture failure after bile duct reconstruction. If intraperitoneal drains placed during or after surgery are effective, biliary leakages are often curable. However, if the bile leak persists, an additional approach for biliary decompression is needed. In the past, percutaneous intraperitoneal drainage, percutaneous transhepatic biliary drainage, and surgical treatment for biliary leakage were often performed as the first choice, but the usefulness of endoscopic transpapillary drainage for postoperative biliary leakage has been reported.^1^, ^2^, ^3^, ^4^ In the International Study Group of Liver Surgery criteria,^2^ Grade B bile leak is defined as “bile leak requiring treatment other than relaparotomy” and Grade C as “bile leak requiring relaparotomy”. According to these criteria, endoscopic treatment is also the recommended method of drainage for postoperative grade B or C biliary leakage with abdominal pain and/or infection.

The efficacy of endoscopic biliary decompression for post‐cholecystectomy biliary leakage has been reported to be 87%–100%,^4^ and transpapillary approaches are usually chosen. Biliary decompression by endoscopic sphincterotomy (EST) alone has also been reported to be effective in several single‐center retrospective studies.^5^, ^6^ In 2 multicenter randomized controlled trials, there were no differences in efficacy for biliary leakage according to stent diameter (7 Fr. vs. 10 Fr.).^7^, ^8^ In patients with post‐cholecystectomy biliary leakage, it is important to relieve biliary pressure regardless of the method used. Biliary leakage is a common complication after central bisegmentectomy, left hepatic trisegmentectomy, and anterior segmentectomy exposing Glisson's capsule.^9^ A previous report showed that the cure rate with endoscopic treatment alone is 49.0%,^3^ but there are few clinical studies focusing on biliary leakage after hepatectomy compared to leakage after cholecystectomy. In previous reports, factors associated with difficulties in endoscopic curative treatment were the grade^4^, ^10^ and the location^3^, ^11^ of the bile leak in both post‐cholecystectomy and post‐hepatectomy biliary leakage. In post‐cholecystectomy biliary leakage, the cure rate was 100% for bile leaks from the cystic duct and Luschka's duct but 40% for those from other sites.^11^ In post‐hepatectomy biliary leakage, the curative rates were higher in cases with bile injuries of the peripheral and hepatic ducts than in those with common bile duct injuries.^3^ In addition, it was reported that disconnected bile duct injuries were often refractory in both post‐cholecystectomy and post‐hepatectomy leakages, but they were easily cured when the leakage point was bridged with a stent.^3^, ^12^, ^13^

In postoperative biliary leakage, in addition to percutaneous intraperitoneal drains placed at the time of surgery, biliary decompression is often performed with external drains such as percutaneous transhepatic biliary drainage or endoscopic nasobiliary drainage (ENBD). External drains are useful procedures for biliary leakages but affect patient quality of life and prolong hospital stay. There are no reports evaluating the usefulness of endoscopic treatment focusing on whether external drains can be removed by endoscopic interventions. In this study, we evaluated the success rates of removal of external drains and all drains after endoscopic transpapillary drainage in post‐hepatectomy and post‐cholecystectomy biliary leakages, respectively. The aim was to clarify the efficacy of endoscopic transpapillary drainage for postoperative biliary leakage and the factors associated with the successful removal of external drains.

## METHODS

### Patients

**FIGURE 1:**
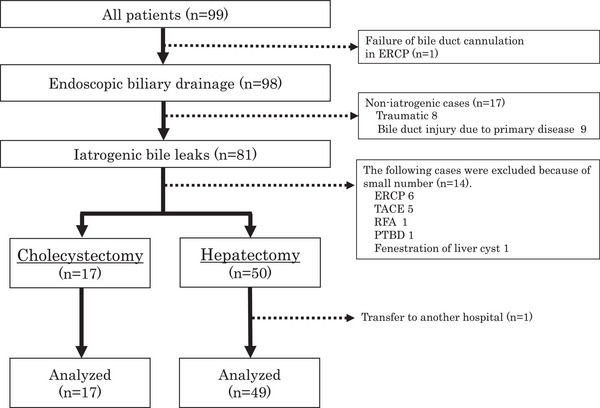
Flow diagram of the study. Of the 99 cases enrolled, 98 had successful endoscopic transpapillary drainage. Of these 98 cases, 67 cases of biliary fistula developed after surgery (17 after cholecystectomy, 50 after hepatectomy). Because one patient was transferred to another hospital after hepatectomy, 17 patients after cholecystectomy and 49 patients after hepatectomy were included in this analysis.

This was a multicenter retrospective study, and 99 patients with bile duct leakage who were initially treated with endoscopic biliary procedures were enrolled at Osaka University Hospital and 12 affiliated hospitals from April 2014 to March 2019. Patients eligible for the study were identified from the database of each hospital, and the necessary data were collected from the medical records. In this study, the following cases were excluded: one patient with difficulty in bile duct cannulation and 17 patients in whom the cause of bile leakage was not iatrogenic (trauma and bile duct injury due to primary disease, Figure 1). Among 81 patients with iatrogenic bile leak, 14 were excluded because of the small number of related cases (Figure 1). A total of 66 patients who underwent surgery were included in this study (cholecystectomy, 17 patients; and hepatectomy, 49 patients). This study was approved by the Institutional Review Board of Osaka University (No. 19436) and was conducted in accordance with the Declaration of Helsinki. Written informed consent was waived because participants were offered the opportunity to withdraw from the study.

### Endoscopic treatment

Endoscopic transpapillary drainage was generally used as the first choice for the treatment of postoperative biliary leakage in this study. ERCP was performed using a JF‐260 V or TJF‐260 V endoscope (Olympus). In many cases, midazolam and pentazocine were used for sedation during treatment. The cases included in this study were supervised by each participating facility's instructor of the Japan Gastroenterological Endoscopy Society. The need for EST and the number, diameter, and drainage method were judged by the operators.

### Definitions

Postoperative biliary leakage was determined by the nature of contents and/or drainage volume of the external drain placed at the time of surgery or by cholangiography at the time of ERCP after suspicion of bile leakage based on clinical symptoms and/or computed tomography. The success of the initial endoscopic transpapillary drainage was defined as follows: the cases in which the bile leak from the percutaneous external tube disappeared or the persistent bile leak on imaging studies was improved after initial endoscopic interventions.

**FIGURE 2:**
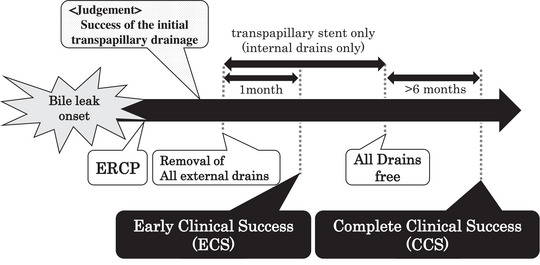
Definitions of the primary endpoints. Early clinical success (ECS) was defined as any case in which all the external drains, including the endoscopic nasobiliary drainage (ENBD) tube, were successfully removed after endoscopic drainage with no recurrence of bile leaks for 1 month. Complete clinical success (CCS) was defined as any case in which all internal biliary drainage tubes were removed with no recurrence of bile leaks for 6 months after the achievement of ECS.

Early clinical success (ECS) was defined as follows: all the external biliary drainage tubes, including the ENBD tube, were successfully removed after the endoscopic intervention, and there was no recurrence of bile leakage for 1 month (Figure 2). Complete clinical success (CCS) was defined as follows: in the cases that had achieved ECS, all the internal biliary drainage tubes were removed, and there was no recurrence of bile leakage for 6 months (Figure 2). The rates of ECS and CCS cases were defined as the ECSR and CCSR, respectively.

Identification of the location and type of bile duct injury was determined by the site of leakage on cholangiography at the time of ERCP. The bile ducts were classified according to five locations that describe the site of bile leakage: common bile duct, cystic duct, the first branch of the intrahepatic bile duct, second branch, and third and/or higher branches. The types of bile duct injuries were classified into three patterns: peripheral type, completely disconnected type, and partially disconnected type. The peripheral type was defined as a case in which bile leakage from the peripheral branches was identified in the liver or cystic duct due to surgery. In disconnected types of bile duct injury, the bile duct was divided at the site of injury; the completely disconnected type was defined as a case in which the bile duct wall was not continuous due to injury, and the partially disconnected type was defined as a case other than the completely disconnected type. The positions of the transpapillary deployment of plastic stent, ENBD tube, or self‐expandable metal stent (SEMS) were classified into three patterns: near leakage, bridge, and intracavity. Near leakage was defined as a case where the drainage tube was placed near the bile duct leakage. Bridging was defined as the deployment of the drainage tube covering the bile duct leakage. Intracavity was defined when the tip of the drainage tube was placed in the leak cavity. As an endoscopic transpapillary drainage method, bridging was the basic approach for the disconnected type, but in cases of difficulty, placement near the leak was performed. In the peripheral type, a drainage tube was placed near the leak point or in the cavity. Biloma is a secondary cyst that collects bile in the subserosal space of the liver and is usually caused by injury to the liver and the bile duct due to trauma or surgery.^14^

### Data collection

The following data were obtained from the medical records at each institution: (i) patient characteristics (sex, age, body mass index (BMI), primary disease, surgical treatment, and comorbidities); (ii) information on damaged bile ducts (location of leak and type of injury); (iii) information on bile leak cavity (biloma, infection, and percutaneous drainage); (iv) information on initial endoscopic transpapillary drainage (number of drain tubes, method (plastic stent, self‐expandable metal stent, or ENBD), diameter and position of drains); (v) information on EST and ERCP‐related complications (cholangitis, pancreatitis, bleeding)^15^; and (vi) patient outcomes.

### Outcome measures

The primary endpoint of this study was to evaluate the ECSR and CCSR after endoscopic transpapillary procedures in patients with postoperative biliary leakage. The secondary endpoints were to investigate the factors associated with ECS in postoperative biliary leakage.

### Statistical analysis

Data are presented as medians with interquartile ranges unless otherwise noted. The Mann‒Whitney U test was used for continuous variables, and the Chi‐square test was used for nominal variables. Factors associated with ECS were examined by logistic regression analysis. In all analyses, a *p*‐value <0.05 was considered indicative of statistical significance. Statistical analyses were performed with JMP software (ver. 16.0.0; SAS Institute Inc.).

## RESULTS

Patient characteristics are shown for 17 patients in the post‐cholecystectomy biliary leakage group (CBL group) and 49 patients in the post‐hepatectomy biliary leakage group (HBL group, Table 1). In the CBL group, cholecystectomy was mainly performed for acute cholecystitis or gallbladder stones (14/17, 82.4%). The reasons for hepatectomy in the HBL group were mostly malignant diseases, with hepatocellular carcinoma in 33 cases (67.3%) and metastatic liver tumor in 11 cases (22.4%). In the CBL group, 11 patients (64.7%) had no comorbidities regarding liver cirrhosis, diabetes mellitus, and other primary cancers, and five patients (29.4%) had diabetes mellitus. On the other hand, 34 patients (69.4%) in the HBL group had one or more comorbidities. In the CBL group, biliary leakage occurred in the extrahepatic bile duct (9/17, 52.9%), including the common bile duct in six cases and the cystic duct in three cases. In the HBL group, biliary leakage occurred in the intrahepatic duct in almost all cases (48/49, 98.0%). External drains were placed at surgery in 58.8%/85.7% of patients in the CBL/HBL group, biloma was observed in 29.4%/79.6%, and infection of the bile leak was a complication in 42.2%/57.1%. The median interval from surgery to first ERCP was 7.0/31.0 days for CBL/HBL patients. ENBD was the most common initial drainage method in both groups. The number of transpapillary drains was one in almost all cases, and the position of the drainage tube was most often near the leak in both groups. EST was performed in 10 patients (58.8%) in the CBL group and 21 patients (42.9%) in the HBL group (Table 1).

**TABLE 1:** Patient characteristics.

	Cholecystectomy	Hepatectomy
	*n* = 17	*n* = 49
Gender, *n*		
Male/female	12/5	39/10
Age, years		
Median (IQR)	76.0 (19.0)	75.0 (9.0)
BMI, kg/m^2^		
Median (IQR)	21.0 (3.8)	21.2 (4.2)
Primary disease, *n* (%)		
Hepatocellular carcinoma	1 (5.9)	33 (67.3)
Liver metastases	‐	11 (22.4)
Intrahepatic cholangiocarcinoma	‐	3 (6.1)
IPNB	‐	1 (2.0)
Angiomyolipoma	‐	1 (2.0)
Acute cholecystitis/GBS	14 (82.4)	‐
Gallbladder cancer	1 (5.9)	‐
Gastric cancer	1 (5.9)	‐
Comorbidity, *n* (%)		
No	11 (64.7)	15 (30.6)
Yes^a^	6 (35.3)	34 (69.4)
Liver cirrhosis	1 (5.9)	16 (32.7)
Diabetes	5 (29.4)	13 (26.5)
Other primary cancer	1 (5.9)	12 (24.5)
External drainage at surgery, *n* (%)	10 (58.8)	42 (85.7)
Leak location, *n* (%)		
Extrahepatic bile duct	9 (52.9)	1 (2.0)
Common bile duct/cystic bile duct	6 (35.3) / 3 (17.6)	1 (2.0) / ‐
Intrahepatic bile duct	7 (41.2)	48 (98.0)
1st branch/2nd /over 3rd	1 (5.9)/2 (11.8)/4 (23.5)	17 (34.7)/18 (36.7)/13 (26.5)
Unknown	1 (5.9)	‐
Type of bile duct injury, *n* (%)		
Completely disconnected type	3 (17.6)	15 (30.6)
Partially disconnected type	8 (47.1)	12 (24.5)
Peripheral type	6 (35.3)	22 (44.9)
Biloma, *n* (%)	5 (29.4)	39 (79.6)
Infection, *n* (%)	7 (41.2)	28 (57.1)
Period from surgery to transpapillary drainage, days		
Median (IQR)	7.0 (11.0)	31.0 (44.0)
Drainage method, *n* (%)		
PS	4 (23.5)	11 (22.4)
SEMS	‐	1 (2.0)
ENBD	13 (76.5)	37 (75.5)
Drain diameter		
≤6Fr./≥7Fr.	6/11	22/27
Number of drains, *n*		
0/1/2	0/17/0	0/46/3
Position of transpapillary tube, *n* (%)		
Bridge	1 (5.9)	6 (12.2)
Near leakage	15 (88.2)	39 (79.6)
Intracavity	1 (5.9)	4 (8.2)
EST, *n* (%)	10 (58.8)	21 (42.9)

^a^ Includes liver cirrhosis, diabetes, and other primary cancer.

Abbreviations: BMI, body mass index; GBS, Gallbladder stones; ENBD, endoscopic nasobiliary drainage; EST, endoscopic sphincterotomy; IPNB, Intraductal papillary neoplasm of a bile duct; IQR, interquartile range; PS, plastic stent; SEMS, self‐expandable metal stent;.

In the CBL group, both successful initial transpapillary drainage and ECS were achieved in all cases without additional treatment, and the ECSR was 100% (Table 2). In seven of 13 patients with ENBD as initial drainage, ENBD was changed to internal stenting. Finally, transpapillary drainage tubes could be removed in 16 of 17 patients, and one patient required continuous placement of a plastic stent due to bile duct stenosis. Ten patients could be followed up for more than half a year, and the CCSR was 90% (9/10). In one case with non‐CCS, the bile leak recurred on day 23 after removal of the internal stent, and ENBD was replaced in the patient, who eventually become stent‐free.

**TABLE 2:** Primary endpoint.

	Cholecystectomy	Hepatectomy
	*n* = 17	*n* = 49
Period from surgery to transpapillary drainage, days, median (IQR)	7.0 (11.0)	31.0 (44.0)
Success rate of initial transpapillary drainage	100% (17/17)	44.9% (22/49)
Early clinical success rate	100% (17/17)	44.9% (22/49)
Period from endoscopic interventions to removal of all external drains, days, median (IQR)	12.0 days (9.3)	28.0 days (81.0)
All drain‐free success rates after ECS	94.1% (16/17)	100% (22/22)
Complete clinical success rate	90.0% (9/10)	100% (19/19)

Abbreviations: ECS, early clinical success; IQR, interquartile range.

In the HBL group, initial transpapillary drainage was successful in 22 patients (44.9%), and two of them had recurrent bile leakage within 1 month after the removal of the external drains. In 27 unsuccessful cases of initial transpapillary drainage, the external drains were finally removed by re‐ERCP, and no recurrence was observed in two cases. Therefore, the ECSR in the HBL group was 44.9% (22/49, Table 2). In 12 cases of the HBL group, internal stents were placed as initial transpapillary drainage, and six of them achieved ECS with initial treatment alone. In all 22 patients that achieved ECS, internal stents were successfully removed. Nineteen patients were followed for more than half a year, and the CCSR was 100% (19/19, Table 2). In the 27 patients who did not achieve ECS, we added percutaneous drainage. Seventeen patients had successful removal of all drains with additional percutaneous drainage, seven died of the primary disease with percutaneous tubes remaining in place, two underwent reoperation, and one was transferred to another hospital without removal of all drains.

Patient characteristics in the HBL group were compared between the ECS‐achieved group (*n* = 22, ECS group) and the non‐ECS group (*n* = 27, Table 3). The median follow‐up was 1018 days and 632 days in the ECS and non‐ECS groups, respectively. There was no difference in the background between the two groups, such as the primary disease, comorbidity, external drainage, and type of bile duct injury. However, patients in the ECS group had a significantly lower BMI than patients in the non‐ECS group (20.3 vs. 22.8 kg/m^2^, *p* < 0.01, Table 3).

**TABLE 3:** Comparison of patient characteristics in the early clinical success and non‐early clinical success groups for bile leaks after hepatectomy.

	ECS group *n* = 22	non‐ECS group *n* = 27	*p*‐value
Gender, *n*			
Male	17	22	0.72
Female	5	5	
Age, years			
Median (IQR)	72.5 (8.8)	76.0 (7.5)	0.20
BMI, kg/m^2^			
Median (IQR)	20.3 (2.9)	22.8 (3.7)	<0.01
Primary disease, *n*			
Hepatocellular carcinoma	16	17	0.61
Liver metastases	4	7	
Intrahepatic cholangiocarcinoma	1	2	
IPNB	0	1	
Angiomyolipoma	1	0	
Comorbidity, *n*			
No	10	5	0.06
Yes^a^	12	22	
Liver cirrhosis	7	9	0.14
Diabetes	4	9	0.23
Other primary cancer	4	8	0.35
External drainage at surgery, *n*			
No	4	3	0.69
Yes	18	24	
Leak location, *n*			
Extrahepatic bile duct	1	0	0.26
Intrahepatic bile duct	21	27	
Type of bile duct injury, *n*			
Completely disconnected type	6	9	0.81
Partially disconnected type	5	7	
Peripheral type	11	11	
Biloma, *n*			
No	2	8	0.08
Yes	20	19	
Infection, *n*			
No	9	12	0.80
Yes	13	15	
Period from surgery to transpapillary drainage, days			
Median (IQR)	31.0 (33.3)	32.0 (57.5)	0.85
Overall observation periods, days			
Median (IQR)	1017.5 (761.5)	632.0 (780.5)	<0.05
Drainage method, *n*			
PS, SEMS	6	6	0.68
ENBD	16	21	
Drain diameter, n			
≤6 Fr.	11	11	0.52
≥7 Fr.	11	16	
Position of transpapillary tube, *n*			
Near leakage	17	22	0.94
Bridge	3	3	
Intracavity	2	2	
EST, *n*			
No	12	16	0.74
Yes	10	11	

^a^ Including any liver cirrhosis, diabetes, and other primary cancer.

Abbreviations: BMI, body mass index; ECS, early clinical success; ENBD, endoscopic nasobiliary drainage; EST, endoscopic sphincterotomy; IPNB, Intraductal papillary neoplasm of bile duct; IQR, interquartile range; PS, plastic stent; SEMS, self‐expandable metal stent.

*p*‐Values were calculated using the Mann‐Whitney U test or Fisher's exact test.

The factors associated with ECS in the HBL group were examined by logistic regression analysis (Table 4). In univariate analysis, BMI < 21.2 kg/m^2^ (median BMI in the HBL group) and no comorbidities were significant factors associated with ECS. Multivariate analysis of these two categories showed that BMI<21.2 kg/m^2^ was a significant factor (odds ratio 0.27, 95% confidence interval 0.07–0.92, *p* = 0.04). EST did not affect the ECSR, ERCP‐related complications such as pancreatitis and cholangitis, or patient outcomes in either the CBL or HBL group (Table 5).

**TABLE 4:** Factors associated with early clinical success in the hepatectomy biliary leakage group.

		Univariate analysis			Multivariate analysis		
	ECSR (%), *n*	OR	95% CI	*p* ‐value	OR	95% CI	*p*‐value
Gender							
Male	43.6 (17/39)	1	0.32–5.37	0.72			
Female	50.0 (5/10)	1.29					
Age, years							
<75.0	54.2 (13/24)	1	0.15–1.50	0.20			
≥75.0	36.0 (9/25)	0.48					
BMI, kg/m^2^							
<21.2	66.7 (16/24)	1	0.05–0.55	<0.01	1	0.05–0.65	<0.01
≥21.2	24.0 (6/25)	0.16			0.18		
Origin of liver tumor							
Primary^a^	47.4 (18/38)	1	0.15–2.47	0.52			
Metastatic	36.4 (4/11)	0.63					
Comorbidity							
Noting	66.7 (10/15)	1	0.07–0.95	0.04	1	0.09–1.41	0.14
Yes^b^	35.3 (12/34)	0.27			0.35		
Liver cirrhosis							
No	45.5 (15/33)	1	0.27–3.11	0.91			
Yes	43.8 (7/16)	0.93					
Diabetes							
No	50.0 (18/36)	1	0.10–1.63	0.23			
Yes	30.8 (4/13)	0.44					
Other primary cancer							
No	48.6 (18/37)	1	0.14–2.06	0.36			
Yes	33.3 (4/12)	0.53					
External drainage at the surgery							
No	57.1 (4/7)	1	0.11–2.83	0.49			
Yes	42.9 (18/42)	0.56					
Leak location							
Central side^c^	33.3 (6/18)	1	0.64–7.13	0.22			
Peripheral side^d^	51.6 (16/31)	2.13					
Type of injury							
Completely disconnected type	40.0 (6/15)	1	ref				
Partially disconnected type	41.7 (5/12)	1.07	0.22–5.10	0.93			
Peripheral type	50.0 (11/22)	1.50	0.40–5.87	0.55			
Biloma							
No	20.0 (2/10)	1	0.91–30.32	0.07			
Yes	51.3 (20/39)	4.21					
Infection							
No	42.9 (9/21)	1	0.37–3.67	0.80			
Yes	46.4 (13/28)	1.16					
Drainage method							
PS, SEMS	50.0 (6/12)	1	0.20–2.86	0.68			
ENBD	43.2 (16/37)	0.76					
Drain diameter							
≤6Fr.	50.0 (11/22)	1	0.22–2.14	0.52			
≥7Fr.	40.7 (11/27)	0.69					
Drain position							
Near leakage	43.6 (17/39)	1	ref				
Bridge	50.0 (3/6)	1.29	0.22–7.78	0.77			
Intracavity	50.0 (2/4)	1.29	0.14–11.70	0.81			
EST							
No	42.9 (12/28)	1	0.38–3.82	0.74			
Yes	47.6 (10/21)	1.21					

^a^ Primary includes hepatocellular cancer, intrahepatic cholangiocarcinoma, intraductal papillary neoplasm of bile duct (IPNB), and angiomyolipoma.

^b^ Including any liver cirrhosis, diabetes, and other primary cancer.

^c^ Central side of the leak location contains the extrahepatic bile duct and 1st branch of the intrahepatic bile duct.

^d^ Peripheral side of the leak location contains 2nd and over 3rd branches of the intrahepatic bile duct.

BMI, body mass index; CI, confidence interval; ECSR, early clinical success rate; ENBD, endoscopic nasobiliary drainage; EST, endoscopic sphincterotomy; OR, odds ratio; PS, plastic stent; SEMS, self‐expandable metal stent.

*p*‐Values were calculated using logistic regression analysis.

**TABLE 5:** Comparative outcomes and complication rates with and without endoscopic sphincterotomy.

		With EST	Non‐EST	*p*‐value
Post‐cholecystectomy (*n*)		10	7	
	ECSR (%)	100	100	1.00
	Period from ERCP to the removal of external drains, days, median (IQR)^a^	8.5 (8.0)	15.5 (3.3)	0.08
	ERCP‐related complications, *n* (%)			
	Cholangitis	0	0	N/A
	Pancreatitis	0	0	N/A
	Bleeding	0	0	N/A
	Recurrence of bile leak, *n* (%)			
	After removing external drains^a^	0	0	N/A
	After removing all drains^b^	1 (10.0)	0	0.39
Post‐hepatectomy (*n*)		21	28	
	ECSR (%)	47.6	42.9	0.59
	Period from ERCP to the removal of external drains, days, median (IQR)^a^	50.5 (69.8)	28.0 (95.5)	0.83
	ERCP‐related complications, *n* (%)			
	Cholangitis	2 (9.5)	1 (3.6)	0.39
	Pancreatitis	1 (4.8)	2 (7.1)	0.73
	Bleeding	1 (4.8)	0	0.24
	Recurrence of bile leak, *n* (%)			
	After removing external drains^a^	1 (9.1)	1 (7.7)	0.84
	After removing all drains^b^	0	0	N/A

^a^ Only cases in which transpapillary drainage was successful and the external drains were removed were counted.

^b^ Only the cases that achieved ECS were counted.

ECS, early clinical success; ECSR, early clinical success rate; ERCP, endoscopic retrograde cholangiopancreatography; EST, endoscopic sphincterotomy; IQR, interquartile range.

*p*‐Values were calculated using the Chi‐squared test.

## DISCUSSION

In this study, we demonstrated the efficacy of endoscopic transpapillary drainage for postoperative biliary leakage by evaluating the clinical success rate after initial endoscopic drainage and the ECSR, which represents the frequency of freedom from external drainage after endoscopic procedures. The success rate of initial drainage was 100% for biliary leakage after cholecystectomy and 44.9% for biliary leakage after hepatectomy (Table 2). The ECSR was 100% for biliary leakage after cholecystectomy and 44.9% for biliary after hepatectomy (Table 2). Almost all patients with ECS achieved all‐drains‐free. Achievement of an external drain‐free status contributed greatly to patients' quality of life and could be one of the predictors of treatment response after endoscopic therapy for postoperative biliary leakage.

In post‐cholecystectomy biliary leakage, ECS was achieved in all patients at initial endoscopic drainage, and bile leakage improved regardless of the drainage method, stent diameter, and with or without EST during the initial endoscopic procedure. This study showed that sustained biliary decompression is important as in previous reports.^5^, ^6^, ^7^, ^8^ Therefore, internal stenting instead of ENBD may be acceptable for biliary leakage after cholecystectomy. On the other hand, in post‐hepatectomy biliary leakage, approximately half of the patients did not achieve ECS. This suggests that, unlike after cholecystectomy, multiple factors other than biliary decompression may be involved in the therapeutic effects. In this study, BMI was significantly higher in the non‐ECS group than in the ECS group. There have been no reports that mentioned the relationship between BMI and the efficacy of endoscopic treatment for post‐hepatectomy biliary leakage. The cutoff for BMI in our study was set at the median of the HBL group, and BMI<21.2 kg/m^2^ was a factor associated with ECS in post‐hepatectomy biliary leakage. An association between BMI and visceral fat type obesity in men was reported in a Japanese health examination cohort,^16^ and it was suggested that patients with high BMI accumulated a large amount of visceral fat. Visceral fat accumulation leads to increased levels of proinflammatory cytokines such as TNF‐α and IL‐6 and decreased levels of anti‐inflammatory cytokines such as adiponectin.^17^ Therefore, the involvement of visceral fat in the persistent inflammatory response is conceivable. The overall observation period was shorter in the non‐ECS group than in the ECS group. Of the 27 patients in the non‐ECS group, seven died with external drains in place after endoscopic treatment, and the cause of death was exacerbation of primary disease in four patients and liver failure in three patients (data not shown). In these seven fatal cases, the median time from surgery to endoscopic intervention was 88 days, and the median time from endoscopic intervention to death was 89 days, including some cases in which endoscopic intervention was performed after the biliary leakage had become refractory (data not shown). Because some cases of post‐hepatectomy biliary leakage are refractory due to obesity or exacerbation of the underlying disease, it is necessary to consider various treatment options from the early onset of bile leakage.

We focused on postoperative biliary leakage in this study, and information on bile duct stricture at the time of endoscopic intervention was not collected, because, in postoperative situations, bile leaks from the transected bile duct are more common than those caused by increased intrabiliary pressure due to bile duct stricture. However, in cases with biliary stricture, persistent elevation of biliary pressure without adequate drainage may lead to refractoriness if the leak point is upstream of the biliary stricture. Some of the refractory cases in our study may be cases in which the biliary stricture contributed to refractoriness. In 15 cases of complete disconnection type of the HBL group, the ECSR was 50.0% (1/2) for “bridging” and 38.5% (5/13) for “near leakage”; in 12 cases of partial disconnection type, 50.0% (2/4) for “bridging” and 37.5% (3/8) for “near leakage” (data not shown). In the disconnected type, bridging across the disconnected part is theoretically desirable.^3^, ^12^, ^13^ If bridging was technically impossible, endoscopic drainage near the leak site may be attempted to decompress the intrabiliary pressure in the partially disconnected type, but the surgical approach should be considered first in the completely disconnected type.

There were no cases of postoperative biliary leakage treated with EST alone as a biliary decompression method, and it was difficult to evaluate the benefit of EST in this study. The risk of retrograde cholangitis was a concern in EST cases, but the condition did not occur during the observation period. The incidence of post‐ERCP pancreatitis (PEP) and bleeding did not differ between cases with and without EST. While EST prevents PEP,^13^ complications such as severe bleeding or perforation may worsen the overall condition. Recently, elderly patients on regular antithrombotic medications have often been encountered, so the need for EST should be discussed on a case‐by‐case basis.

This study has several limitations. Due to the retrospective nature of the study, the selection criteria and the timing of endoscopic treatment varied among the participating hospitals, depending on the operator's discretion. In addition, the study was based on a small number of cases, so it is desirable to increase the number of cases and conduct a prospective study based on a standardized protocol in the future.

In conclusion, biliary leakage after cholecystectomy was improved by a single endoscopic treatment, and biliary decompression by the endoscopic intervention was very effective. On the other hand, in post‐hepatectomy biliary leakage, BMI was a useful predictor of refractory biliary leakage, and approximately half of the cases required multidisciplinary treatment combining adequate percutaneous and endoscopic drainage. Achieving freedom from external drainage contributed greatly to patients’ quality of life and could be one of the predictors of treatment response after endoscopic therapy for postoperative biliary leakage.

## CONFLICT OF INTEREST STATEMENT

None.
